# Role of Oxidative Stress on the Etiology and Pathophysiology of Amyotrophic Lateral Sclerosis (ALS) and Its Relation with the Enteric Nervous System

**DOI:** 10.3390/cimb45040217

**Published:** 2023-04-07

**Authors:** Laura López-Pingarrón, Henrique Almeida, Marisol Soria-Aznar, Marcos C. Reyes-Gonzales, María Pilar Terrón, Joaquín J. García

**Affiliations:** 1 Department of Pharmacology, Physiology and Legal and Forensic Medicine, Faculty of Medicine, University of Zaragoza, 50009 Zaragoza, Spain; msoria@unizar.es (M.S.-A.); mreyesg@unizar.es (M.C.R.-G.); jjgarcia@unizar.es (J.J.G.); 2 i3S—Instituto de Investigação e Inovação em Saúde, Porto University, 4200-135 Porto, Portugal; almeidah@med.up.pt; 3 Department of Biomedicine, Faculty of Medicine, Porto University, 4200-319 Porto, Portugal; 4 Department of Obstetrics and Gynecology, Hospital-CUF Porto, 4100-180 Porto, Portugal; 5 Department of Physiology, Faculty of Medicine and Health Sciences, University of Badajoz, 06006 Badajoz, Spain; pilarts@unex.es

**Keywords:** amyotrophic lateral sclerosis, superoxide dismutase (SOD_1_), neurodegeneration, oxidative stress, excitotoxicity, and neuroinflammation

## Abstract

Amyotrophic lateral sclerosis (ALS) is a progressive neurodegenerative disease affecting motor neurons in the spinal cord, cerebral cortex, and medulla oblongata. Most patients present a clinical phenotype of classic ALS—with predominant atrophy, muscle weakness, and fasciculations—and survival of 3 to 5 years following diagnosis. In the present review, we performed a literature search to provide an update on the etiology and pathophysiological mechanisms involved in ALS. There are two types of ALS: the familial form with genetic involvement, and the sporadic form with a multifactorial origin. ALS pathophysiology is characterized by involvement of multiple processes, including oxidative stress, glutamate excitotoxicity, and neuroinflammation. Moreover, it is proposed that conditioning risk factors affect ALS development, such as susceptibility to neurodegeneration in motor neurons, the intensity of performed physical activity, and intestinal dysbiosis with involvement of the enteric nervous system, which supports the existing theories of disease generation. To improve patients’ prognosis and survival, it is necessary to further deepen our understanding of the etiopathogenesis of ALS.

## 1. Introduction: Relevant Clinical Features of ALS

Amyotrophic lateral sclerosis (ALS) is a progressive neurodegenerative disorder that has devastating effects on motor neurons and exhibits vast disease heterogeneity at the clinical, genetic, and neuropathological levels [1,2]. The term “amyotrophic lateral sclerosis” was coined by the French neurologist Jean-Martin Charcot in the 19th century. “Amyotrophic” refers to the muscular atrophy that characterizes this disease, and “lateral sclerosis” to the scarring or hardening of tissues in the lateral part of the spinal cord. ALS involves the loss of lower motor neurons of the anterior horn of the spinal cord and brainstem nuclei, and the degeneration and loss of pyramidal neurons of the primary motor cortex and corticospinal tracts [1].

In Europe and North America, ALS incidence rates range from 1.5 to 2.7 cases per 100,000 population each year, while prevalence rates range from 2.7 to 7.4 per 100,000 population; however, there are significant geographical variations—for example, Portugal had an ALS prevalence of 10.3 in 2016 [3]. Within the age group with the highest risk of ALS development (45–75 years), the incidence is between 4–8 cases per 100,000 population [4]. To date, few published epidemiological studies describe ALS in Spain. A study including the population of Catalonia reported an incidence of 1.4 and a prevalence of 5.4 per 100,000 inhabitants, based on data collected up to 2011. Another study was conducted in Navarra using more recent data (collected up to 2018), and reported an incidence of 2.47 per 100,000 cases, and a prevalence of 6.64 per 100,000 individuals (95% CI: 4.52–8.45) [5].

ALS onset typically occurs in middle age, between 55–65 years of age. In most cases, it presents as progressive muscle atrophy and weakness. The prognosis is dismal, with a survival of 3–5 years from the onset of symptomatology. The most frequent complications are respiratory failure due to weakness of the thoracic musculature, and aspiration pneumonia due to dysphagia. About 20% of patients survive for 5 years after diagnosis, and 5% for 10 years or more. ALS is more frequent in males (2:1 male-to-female ratio). Compared with women, men have a higher risk of developing sporadic onset ALS, although this risk tends to equalize with increasing age. This progressive disease inexorably leads to death. Patients with genetic or bulbar involvement have a worse prognosis [4].

The specific cause of ALS is currently unknown, but it is believed that disease development is influenced by the association of a diversity of genes and environmental factors [6]. Up to 10% of ALS patients have at least one affected family member and are defined as patients with familial ALS (f-ALS), which has an earlier onset, starting around 40–60 years of age [1]. The remaining 90–95% of cases occur randomly, defined as sporadic ALS (sALS), which has a multifactorial origin [7,8].

Regarding the disease pathogenesis, it has been established that a complex interplay of molecular and cellular processes leads to neurodegeneration. Glutamate excitotoxicity induces cytoplasmic calcium accumulation and increased oxidative stress. Mutations in the *C9ORF72*, *FUS*, TDP-43, and *SOD_1_* genes lead to RNA dysregulation, which results in accumulation of intraneuronal aggregates and defective axonal transport. Additionally, microglia activation and neuroinflammation result in the secretion of proinflammatory cytokines and neurotoxicity, which also determine the neurodegeneration. All of these mechanisms, which will be further described throughout this review, constitute the neuropathological signature of ALS, characterized by the loss of neuromuscular connection, axonal retraction, and subsequent death of upper and lower motor neurons [7,8].

Several theoretical models have been proposed to explain the pathophysiological onset of ALS, and the underlying onset of molecular changes. The so-called “forward death” model proposes that ALS is primarily a disorder of motor neurons in the cortex, which monosynaptically connect with neurons in the anterior horn of the medulla and mediate the anterograde degeneration of motor neurons through glutamate excitotoxicity. Cortical hyperexcitability has been proposed as one key pathophysiological process, and a possible important diagnostic marker in early disease stages. On the other hand, the retrograde degeneration hypothesis proposes that ALS begins within the muscles or at the neuromuscular junction, when noxious factors induced by free radicals are retrogradely transported from the periphery to the neuronal body, where they exert toxic effects. Finally, there is a proposed hypothesis of mixed degeneration, with independent and simultaneous involvement of both upper and lower motor neurons [9].

The most common clinical form is known as spinal ALS, characterized by focal muscle weakness and atrophy, which tends to spread with disease progression. Within months to a few years, all striated muscles are affected, except the intrinsic eye muscles, sphincters, and heart muscle. Weakness most often begins in the distal muscles of the extremities—for example, with a loss of strength in either one hand or one foot. Additionally, due to upper motor neuron involvement, patients will present with spastic hypertonia, hyperreflexia, and Babinski’s sign. ALS does not involve sensory or autonomic system impairment [1,9].

About 25–30% of patients debut with a more specific clinical presentation of primarily bulbar involvement, exhibiting dysarthria, dysphagia, dysphonia, tongue twitching, or, more rarely, masseter weakness. Bulbar onset is more prevalent among females, is highly associated with cognitive compromise and altered emotional expression, and often directly correlates with depression. In spinal ALS, the disease evolution is also accompanied with bulbar disturbance, giving rise to joint alteration or oropharyngeal dysphagia due to cranial nerve involvement [9]. There is insufficient scientific evidence to diagnose the onset of the bulbar form in some patients [10].

There are other clinical phenotypes, such as primary lateral sclerosis, in which the affection is limited to the upper motor neurons. In most patients, symptoms begin in the bulbar muscles and arms, followed by leg involvement. It is controversial whether this phenotype truly includes lower motor neuron involvement, but the progression is slower and less aggressive compared with spinal ALS. On the other hand, progressive muscle atrophy can result from predominant involvement of lower motor neurons. This phenotype can begin in any region of the body, has a higher incidence among men, and generally exhibits a delayed onset. Approximately 30% of patients develop upper motor neuron symptoms within 18 months after disease onset [9,10,11].

Although motor symptoms are the most important, half of patients with ALS will suffer extra-motor manifestations to some degree. Behavioral changes or frontotemporal cognitive deficits occur in 35–40% of cases, and frontotemporal dementia (FTD) appears in 10%. FTD is characterized by degeneration of the anterior temporal and frontal lobes, clinically presenting as behavioral changes and impairments of executive functioning and language. ALS and FTD are now considered the two ends of a spectrum, due to the overlapping molecular mechanisms underlying both neurodegenerative disorders [1,11].

ALS is clinically diagnosed, and there is not yet any pathognomonic test. Complementary tests contribute to confirming diagnostic suspicion and to excluding other pathologies. Currently, establishing diagnostic certainty requires the involvement of both motor neurons, or involvement of the lower motor neurons in at least two of four regions (bulbar, cervical, thoracic, or lumbosacral), as well as the exclusion of other etiological conditions [12].

A definitive diagnosis still requires a clinical history, neurological examination, and complementary tests. The most important test is the electromyogram (EMG), which may reveal evidence of a mild decrease in motor conduction, with reduced action potential amplitude, and acute or ongoing muscle denervation, as indicated by the presence of fibrillation potentials and fasciculations present in multiple muscles in the examined regions. Although EMG findings allow distinguishing neurogenic atrophies from muscle diseases, some myopathic processes can produce confusion with ALS, such as chronic polymyositis, Pompe disease, or multifocal motor neuropathy with conduction blocks. Genetic testing has not traditionally been a routine part of evaluation for ALS. If a family history is present, genetic testing could be considered for presymptomatic diagnosis, although the indication for this test is controversial, as it is currently intended only for research trials [12].

Due to its multifactorial origin, there is not yet any effective or etiological treatment available for ALS. Advanced pharmacological trials focused on different mechanisms of action, such as methylcobalamin (to avoid oxidative stress), arimoclomol (directed to decrease autophagy), masitinib (a tyrosine kinase inhibitor for avoiding the neuroinflammation), tauroursodeoxycholic acid (an antiapoptotic agent that inhibits caspase-3), levosimendan (with action on troponin C), or gene therapy with tofersen (BIIB067) (to reduce protein level of SOD_1_), are promising drugs that are under evaluation [13]. Currently, Rilutek^®^ (with the glutaminergic inhibitor riluzole as the active ingredient) seems to extend the survival of ALS patients by about 3 months [13], and edaravone (a neuroprotective antioxidant and mitochondria-acting agent approved in several countries of Asia, as well as USA, Canada, and Switzerland), are the only available drugs that slightly delay the clinical course [13,14]. Based on the role of oxidative stress in ALS pathogenesis, antioxidant drugs have been tested to delay the onset of symptoms, such as vitamin E, vitamin C, carotenes, flavonoids, resveratrol, turmeric, and melatonin [13].

The cornerstone of disease management for ALS patients is still multidisciplinary care for symptomatology control, and supportive measures that improve quality of life. Among the symptoms, spasticity can be treated with baclofen, tizanidine, and muscle stretching. Muscle cramps may respond to magnesium supplements, gabapentin, or carbamazepine. Selective serotonin reuptake inhibitors, amitriptyline, and benzodiazepines may be used for emotional lability. Dietary changes may help to improve nutrition, and a gastrostomy tube is a frequent option for palliative treatment in patients with insufficient caloric intake, or when swallowing becomes dangerous.

One cause of mortality in ALS is respiratory failure due to the loss of motor neurons that innervate respiratory muscles, such as the diaphragm. In such cases of respiratory failure, non-invasive mechanical ventilation is the life-prolonging treatment of choice; additionally, it is sometimes necessary to perform a tracheotomy [15]. Treatments require individual assessments and frequent multidisciplinary interventions, and patients and their families must have an awareness of the conditions [1,16].

Amyotrophic lateral sclerosis is a progressive degenerative neuromuscular disease with a devastating prognosis. Since the origin and exact cause of this disease are not currently known, there are no etiological treatments, and these patients receive only symptomatic treatments and life-support measures. It is essential to continue investigating ALS etiology and pathophysiological mechanisms in order to guide new therapeutic approaches to improve survival and symptom control. In this review, we provide updates on the current proposed causes of ALS onset, delving into the genetic and environmental factors and pathophysiological mechanisms that influence motor neuron neurodegeneration.

Furthermore, we present the possible risk factors that may explain the onset of ALS and its transmission to other motor neurons, such as neurodegeneration, type of physical exercise practiced, or intestinal dysbiosis. Neurodegeneration in ALS occurs more rapidly in motor neurons due to their membrane characteristics and their high energy content, which make them more vulnerable to processes such as free radical damage or glutamate excitotoxicity. At the onset of ALS, the motor neurons that are most susceptible to neurodegeneration are those that innervate type II muscle fibers that fatigue rapidly. The metabolism of these muscle cells can be posited as a determinant in the genesis of oxidative stress and the consequent neuroinflammation that eventually leads to neuronal degeneration.

Additionally, we consider whether the influence of genetic predisposition and the type of physical activity practiced may pose a greater risk for ALS development. We assess the currently available data regarding the relationship of ALS with the intestinal microbiota. Finally, we discuss whether the genetic, environmental, and molecular etiologies of ALS influence the integrity of the enteric nervous system, proposing this as a decisive element in ALS diagnosis and prevention of the onset of the disease.

## 2. Etiology of Amyotrophic Lateral Sclerosis

The etiology of ALS is completely unknown. It has been proposed that it has a multifactorial origin, with an association of genetic and environmental factors involved in disease development [17]. Based on genetic factors, ALS is classified into two forms. Familiar ALS accounts for 10% of the clinical forms, and its pathogenesis originates exclusively due to genetic alterations. On the other hand, the vast majority of ALS cases are considered to be sporadic, and the cause of disease onset is unknown, although it is proposed that it is due to an environment–gene interaction that influences motor neuron degeneration [18].

### 2.1. Familiar Form

Familial ALS is hereditary, with predominance of autosomal dominant forms. Autosomal recessive or X-linked forms have also been described but constitute an insignificant minority of cases. The main risk factor for this type of ALS is a family history, and the developmental triggers are genetic factors. Mutations have been described in over 50 different genes. The most studied mutations are in the *superoxide dismutase* (*SOD_1_*) gene, TDP-43 (RNA binding protein), *gene 9* (*C9ORF72*), and *fused protein in sarcoma* (*FUS*), which account for 75% of mutations in familial cases. The most frequent mutation is the *C9ORF72* gene mutation which is present in 45–50% of familial cases [2].

The first genetic alteration described was a mutation in the Cu/Zn-associated *superoxide dismutase* gene (*SOD_1_*) on chromosome 21. About 20% of familial variants of ALS are related to this mutation, as well as 1–2% of the sporadic form [19]. The cytoplasmic enzyme SOD_1_ has an antioxidant action and is heavily involved in the body’s defense against harmful oxidative effects. SOD_1_ is controlled through its sensitivity to oxygen pressure in tissues, which stimulates its activity upon physical exercise and various chemical compounds. Immunohistochemical analyses have demonstrated that SOD_1_ is abundantly distributed in motor neurons, interneurons, and sensory neurons of the spinal cord. A mutation in this enzyme could induce neurodegeneration due to the accumulation of free radicals in motor neurons, causing their death. Alterations in this enzyme trigger cellular processes related to ALS pathogenesis, such as increased oxidative stress, neuroinflammation, and mitochondrial dysfunction, leading to alterations in the lipid layer of membranes, proteins, and DNA of motor neurons [20]. Patients with mutant *SOD_1_* ALS exhibit more severe lower motor neuron degeneration compared with upper motor neuron degeneration. There appears to be a greater burden of mutated ubiquitinated SOD protein accumulation in the lower motor neurons, and greater axon loss.

In both sporadic ALS and familial ALS, low percentages of ubiquitinated TDP-43 inclusions have been detected in the cytoplasm of neurons. TDP-43 is a heterogeneous nuclear protein responsible for mRNA stability, processing, transport, and translation. Under normal conditions, TDP-43 is expressed in many tissues, including in the nuclei of neurons and glial cells. Mutations in this gene cause a loss of nuclear TDP-43, and formation of pathological aggregates in the cytoplasm, leading to neurodegeneration. Notably, this process is observed not only in ALS, but also in other neurodegenerative diseases, such as Alzheimer’s disease, Lewy body disease, and frontotemporal dementia, implying that this cytoplasmic accumulation may be related to aging and the associated functional loss [21].

Another genetic mechanism involved in ALS involves expanded short hexanucleotide sequence repeats (GGGGCC) in the non-coding region of the *C9ORF72* gene. This mutation has the characteristics of TDP-43 proteinopathy, and aggregates of p62 protein are also produced in the neuronal cytoplasm. The p62 protein is involved in both the proteasomal pathway and autophagy, and there has been growing interest in understanding how these pathways are involved in neurodegeneration. Most familial forms present with this type of mutation [22].

Genetic alteration in the gene for the FUS protein is detected in 3% of familial forms, and 1% of sporadic forms of ALS. This neuropathological subtype is characterized by basophilic inclusions in the cytoplasm of neurons of the motor cortex and spinal anterior horn. Although it is unknown how mutations in FUS cause motor neuron death, it may represent a loss of function of FUS in the nucleus, or an acquired toxic function of mutant proteins in the cytosol [2].

Other gene mutations include that of vascular endothelial growth factor (VEGF). The initial disease trigger could involve a variation in local blood flow, producing an untimely or misplaced vascular hypoperfusion event that triggers a molecular pathology mediated by “angioneurins”, such as VEGF. In addition to its direct neuroprotective effect, VEGF also has an indirect effect that maintains blood flow in the spinal cord and brain at optimal levels. Reduced VEGF could lead to a worse response to hypoxia, and thereby to neuronal degeneration [23].

Most of these genetic factors have been studied in European populations. In future work, it will be important to expand these studies to populations in other countries [18].

### 2.2. Sporadic Form

Sporadic ALS (90% of ALS cases) is not related to family history but appears randomly in patients. Since no specific triggers are known for this type of disease, it is considered to have a multifactorial origin involving the interaction of environmental factors on genetic, immunological, and neuronal susceptibility. In fact, the above-mentioned genetic factors (*SOD_1_* and *C9ORF72*) have been described in 15% of sporadic ALS cases. In this setting, the genetic alterations do not trigger the disease, but rather represent susceptibility to interact with extrinsic factors in the generation of this aggressive disorder [24].

Advanced age, male sex, and family history have been established as verified risk factors. On the other hand, the toxicities of certain chemical substances contained in pesticides, metals, or cigarette smoke have been proposed as predisposing factors in the generation of neuronal damage and loss. Additionally, certain electromagnetic waves can lead to motor neuron death. Another theory involves viral infections—for example, by the human retrovirus K (HERV-K), also regulated by the TDP-43 protein, which induces cell toxicity [25].

Current research raises the possibility of physical activity as a risk factor, as it has been found that many ALS patients [26,27] have certain professions in common, such as being firefighters, military personnel, and athletes. It is also being investigated whether gut microbiota may be related to the onset of ALS, and whether involvement of the enteric nervous system via the gut–brain axis may trigger the disease [28].

These possible risk factors must be further analyzed to clarify our understanding of the cause and origin of ALS [17,25].

## 3. Pathophysiological Mechanisms of the Disease

**Figure 1:**
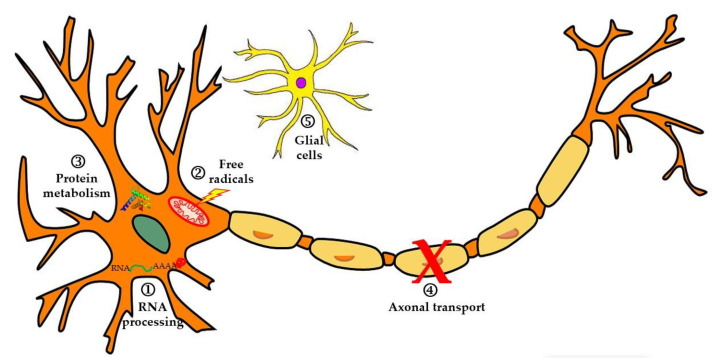
In this image, some of the most relevant pathophysiological mechanisms in ALS are described. 1. Alterations in RNA and RNA-binding proteins such as TDP-43 or FUS, leading to aberrant cytoplasmic aggregates. 2. Increased free radicals that damage the motor neuron membrane, DNA, and mitochondria. 3. Alterations in protein degradation. 4. Defects in axonal transport. 5. Neuroinflammation of microglia that influences the progression of neurodegeneration (figure modified from [19]).

Several cellular and molecular processes underlie ALS development and lead to neurodegeneration and subsequent death of motor neurons (Figure 1). ALS pathophysiology is characterized by alterations at the level of RNA processing, with the appearance of aberrant and toxic RNAs that lose their functions. The disease also involves dysfunction in protein metabolism, inhibition of the ubiquitin-proteasome system (SUP), hyperactivation of autophagy, and disorders in proteins involved in axonal transport. On the other hand, it is considered that neurodegeneration occurs due to high levels of oxidative stress and difficulties in the elimination of free radicals, or through excitotoxicity produced by glutamate. Finally, alterations have been reported at the glial cell level (neuroinflammation), which affect motor neurons and lead to their degeneration [18].

### 3.1. Alterations in RNA and Pathologic Cytoplasmic Aggregates

In ALS, alterations occur in nucleic acid processing, leading to losses of their functions and pathological protein clusters in the neuronal cytoplasm. The first finding indicating the involvement of these processes was the survival gene *MN1*, which is responsible for messenger pre-mRNAs and the axonal transport of messenger RNAs to the motor plate area. Subsequently, the identification of mutations in *TDP43* and *FUS* confirmed that aberrant RNA metabolism could contribute to ALS pathogenesis [18]. Mutations in the *TARDBP* gene encoding the TDP43 protein are a rare cause of familial ALS, but cytoplasmic inclusions of this protein are evident in motor neurons in both the familial and sporadic forms. This protein is predominantly found in the nucleus and interacts with DNA and RNA molecules, intervening in RNA transcription, splicing, and subsequent transport. It remains unclear how mutations in this protein lead to ALS development. Under stress conditions, TDP43 functions by aggregating non-essential RNA in the cytoplasm, such that only essential RNA works. Mutations may prevent this protein from performing its transcription and RNA processing functions, and also lead to the formation of pathological aggregates in the cytoplasm of motor neurons [18].

Another gene related to ALS pathophysiology, and to RNA processing, is the *FUS* protein gene, involved in transcriptional regulation, DNA and RNA processing, and transport of messenger RNAs. Like the TDP43 protein, mutated *FUS* will form stress granules in the cytoplasm and will be unable to properly function. Finally, mutation in the *C9ORF72* gene leads to altered RNA maturation, producing a loss of function and toxic gain, which leads to aberrant aggregates in both the cerebral cortex and spinal cord [26]. These protein clusters are described in inherited forms, and also seen in cases of sporadic ALS. They are an important part of the pathophysiological signature of ALS, in which altered RNA results in the loss of protein functions.

### 3.2. Changes in the Protein Degradation System

Neurodegenerative diseases are characterized by altered protein synthesis by the endoplasmic reticulum (ER), and by alterations in the ubiquitin-proteasome system (SUP) responsible for protein degradation. ALS involves the production of cytoplasmic aggregates of misfolded or aberrant proteins, which induce damaging oxidative stress in the motor neurons, affecting neuronal death. Under certain cellular stress conditions, the endoplasmic reticulum reacts with protein misfolding, as seen in mouse models and ALS patients. The ER comprises ribosomes that are controlled by the nucleolus. ALS patients exhibit enlargement of the nucleolus, to increase ribosomal gene synthesis under stress, and to prevent the accumulation of altered proteins [18].

The pathophysiology of ALS also involves disorders of the SUP protein degradation system and autophagy. These alterations lead to the loss of neuronal homeostasis. In the motor neurons of the anterior horn of the medulla, ALS patients display accumulation of a protein called ubiquilin-2, which is responsible for protein degradation. Aberrant packaging and dysfunction of this protein are observed in ALS patients with mutations for ubiquilin-2, and in ALS patients without mutation in advanced stages of neuronal degeneration. Reports also describe alterations in chaperones, such as valosin, which are responsible for processes such as proteostasis. These changes induce the accumulation of aberrant proteins and generation of cellular stress [28].

### 3.3. Importance of Oxidative Stress and Mitochondrial Dysfunction

**Figure 2:**
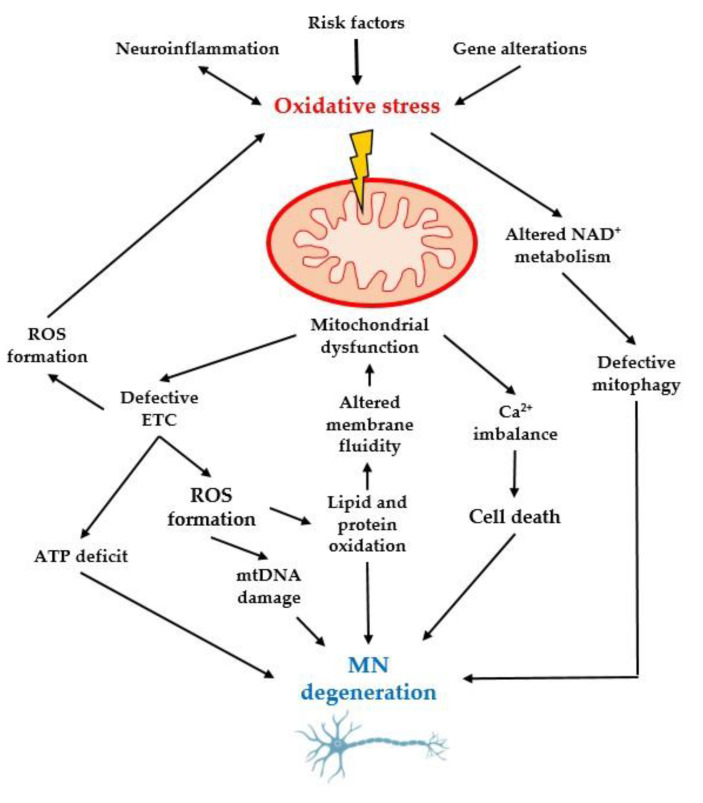
Oxidative stress, together with genetic alterations and neuroinflammation, damages mitochondrial dynamics by altering the redox balance, DNA, and calcium and protein balance, leading to neurodegeneration of MNs [30]. MN: motor neurons; ROS: reactive oxygen species; NAD: Nicotinamide adenine dinucleotide; ATP: Adenosine triphosphate; ETC: electron transport chain.

Oxidative stress has been proposed as an initial factor in the pathogenic development of ALS. This occurs when the rate of free radical production exceeds the motor neurons’ antioxidant capacity. This imbalance between free radical generation and the capacity to scavenge them leads to alterations of motor neuron membrane integrity due to lipid peroxidation, mitochondrial dysfunction, alterations in protein and DNA processing, and excitotoxicity, resulting in neuronal death (Figure 2).

At the intracellular level, most free radicals are generated by the mitochondrial respiratory chain. Thus, alterations in mitochondrial function also contribute to the pathophysiological mechanisms of ALS. Mitochondrial damage aggravates free radical production and lipid peroxidation, causing membrane disorganization, decreased ATP synthesis, and impaired DNA repair, and thus further weakening mitochondrial function [28].

In familial forms of ALS, the discovery of variants in the SOD_1_ enzyme gene supported the hypothesis that free radical toxicity is involved in the process of motor neuron degeneration. The SOD_1_ enzyme catalyzes the conversion of toxic superoxide radicals into oxygen (O_2_) and hydrogen peroxide (H_2_O_2_), such that it has antioxidant capacity in the aerobic metabolism of motor neurons [18]. A mutated SOD_1_ protein could lose its antioxidant capacity and exacerbate oxidative damage through an increase in prooxidant pathways, thereby elevating the oxidative stress exposure of motor neurons. In transgenic mice and human cell lines, abnormal SOD_1_ directly stimulates NADPH oxidase, causing overproduction of reactive oxygen species [29].

Notably, dysfunction of the SOD_1_ enzyme is also observed in sporadic forms of ALS. It has been proposed that the acquisition of oxidative stress by other mechanisms (e.g., through exposure to tobacco or pesticides) may lead to posttranslational modifications of the wild-type SOD_1_ protein, causing it to misfold and acquire toxic properties similar to those of mutant SOD_1_. This mechanism can be considered a major trigger for the pathophysiology of most cases of classical ALS, which lack pathogenic SOD_1_ variants, and likely plays a key role in the devastating progression of the disease [7]. The altered SOD_1_ enzyme is not only present in motor neurons, but also in the surrounding astrocytes and microglia, where it influences the increase of free radicals and continued damage to motor neurons [28].

It has also been proposed that alterations in RNA binding proteins, such as TDP-43 and FUS, may be related to mitochondrial dysfunction, which further promotes the accumulation of free radicals and the maintenance of high oxidative stress levels [6]. Based on this molecular process, various antioxidants have been proposed as potential therapeutic agents. For example, melatonin has antioxidant capacity and can decrease oxidative stress levels [28]. This neuroendocrine hormone is mainly synthesized in the pineal gland and exhibits a wide range of biological functions, such as regulation of the wake-sleep cycle and of metabolism, as well as antioxidant, anti-aging, and anti-tumor effects. Melatonin can prevent cell death, reduce inflammation, stimulate antioxidant enzymes (e.g., SOD_1_), and block calcium channels involved in glutamate excitotoxicity [31]. Several limited studies propose melatonin as a new treatment to combat oxidative stress and excitotoxicity in ALS. The antioxidant capacity of melatonin has been studied in mice with the *SOD_1_* mutation and in patients with sporadic ALS, demonstrating a disease reduction of up to 25% in mice, with a prolonged duration of survival from the onset of symptoms, compared with controls [31]. However, further trials are needed to prove the benefits of melatonin and other antioxidant molecules in ALS.

### 3.4. Axonal Transport Defects in Motor Neurons

From the early stages of ALS, patients exhibit alterations in both retrograde and anterograde axonal transport. Initially, it was thought that neuronal retraction occurred due to mitochondrial dysfunctions or the accumulation of aberrant proteins; however, current evidence reveals mutations in genes that directly encode microtubules of the axon skeleton of motor neurons [18]. Mutations in the *profilin 1* (*PFN1*) gene have also recently been described. *PFN1* is essential for the polymerization of actin filaments, such that mutations in this gene lead to inhibition of axonal growth, increasing the tendency of axonal retraction [32]. Alterations in the dynactin gene, which is responsible for retrograde axonal transport, have also been proposed as a possible cause of motor neuron neurodegeneration [30].

### 3.5. Glutamate Excitotoxicity and Its Relation to Apoptosis

Findings of excess glutamate support the theoretical hypothesis of anterograde disease generation, which postulates that excessive glutamate levels initiate a biochemical cascade leading to motor neuron death. It remains controversial whether glutamate excitotoxicity is a primary pathogenic mechanism, or a secondary effect of altered motor neurons leading to increased glutamate concentration in the postsynaptic cleft. Glutamate is the most abundant excitatory neurotransmitter in the central nervous system. It is released from presynaptic neurons into the cleft, resulting in the activation of glutamate receptors that mediate the entry of calcium and sodium into postsynaptic neurons, causing their depolarization. Subsequently, glutamate is removed from the synaptic cleft through excitatory amino acid transporters, in a process that is highly regulated by neurons and glial cells to avoid excitability and toxicity of motor neurons.

Elevated levels of oxidative stress and alterations in mitochondrial dynamics result in a decreased amount of ATP, which leads to altered sodium levels, causing depolarization and sustained neuronal excitability. Motor neuron hyperexcitability allows an increased influx of cytoplasmic calcium and causes mitochondrial dysfunction, impairment of the respiratory chain, activation of NO synthases (NOS), and the generation of toxic radicals. In addition, it is well-known that excitotoxicity by glutamate includes the generation of reactive oxygen species (ROS) [33]. Excess glutamate aggravates the increases of cytoplasmic calcium and neuronal excitability. Increased calcium leads to the activation of cytoplasmic lytic enzymes that damage the nucleus and membrane structures. In fact, a defect in AMPA-type glutamate receptor editing, leading to enhanced Ca^2+^ permeability, has been reported for a subset of ALS patients [33].

Motor hyperexcitability, and even elevated glutamate levels in the cerebrospinal fluid, have been demonstrated in cases of ALS [34]. Hyperexcitability of the upper motor neurons is established in ALS, first affected by glutamate accumulation and cytoplasmic calcium elevation, with progression to the lower motor neurons. Glutamate accumulation may occur due to abnormal activation of glutamate receptors, which causes excessive Ca^2+^ entry into the postsynaptic neurons and leads to extreme neuronal firing. Glutamate excitotoxicity is thought to result from defective glutamate uptake and transport mechanisms, leading to excessive cytoplasmic Ca^2+^, aberrant Ca^2+^ homeostasis, subsequent mitochondrial dysfunction, and increased free radical production. On the other hand, it has been proposed that aberrant glutamate maintenance in the cleft occurs due to dysfunctional astrocytes and microglia that cannot uptake the surplus. The increased calcium levels, which would be eliminated by the mitochondria under physiological conditions, remain high in the cytoplasm due to the altered mitochondrial dynamics [7]. Overstimulation of motor neurons mediated by excitatory amino acids, such as glutamate, results in abnormal secretion of acetylcholinesterase, which decreases the acetylcholine present in the synaptic cleft, a mechanism that could underlie the loss of muscle strength observed in ALS patients [35].

The best evidence that excitotoxicity is involved in ALS pathogenesis is the fact that riluzole is the only treatment currently available for this disease. In advanced stages of ALS, riluzole increases survival by two to three months, with few side effects. Several pathways have been postulated to explain its mechanism of action, such as inhibition of glutamate release, blockade of glutamate receptors, and blockade of voltage-dependent sodium receptors in motor neurons [36].

It should also be noted that apoptosis may be a late pathway in motor neuron degeneration. Excess free radicals and cytoplasmic calcium can activate pathways of apoptosis and cell death. Although controversial, in mouse studies, the genetic elimination of mitochondrial apoptosis resulted in reduced neuronal loss and improved ALS symptom onset and survival [37].

### 3.6. Roles of Microglia and Neuroinflammation in ALS

Neuroinflammation is a term that broadly describes the reactions of glial cells (astrocytes and microglia) and circulating immune cells (monocytes, neutrophils, and lymphocytes) that interact with nerve cells of the central nervous system in the context of infection, injury, or degeneration. If these cells cannot eliminate the damage, they remain reactive and continue to recruit astrocytes and microglia, leading to a continuous inflammatory process [38,39]. ALS pathogenesis is characterized by motor neuron loss and alterations, but also involves an inflammatory response that is known as neuroinflammation by neighboring cells—in this case, astrocytes and microglia. Pathological studies have revealed proliferation of glial cells and astrocytes in ALS-affected areas. This inflammatory response also influences disease progression and motor neuron degeneration. In both patients with mutant SOD_1_ and patients with sporadic ALS, astrocytes secrete toxic substances, such as proinflammatory interleukins (TNFα and IL1β) and nitric oxide, which damage motor neurons [40]. In addition, the results of a study in an animal model of experimental autoimmune gray matter disease provided evidence for microglial activation with inflammation-mediated toxicity by TNF-α and IL-1, and upper and lower neuronal damage. The activation of p38MAPK signal pathway was present in the development of the motor neuron degeneration [41]. In the early stages of ALS, microglia are activated, and can acquire toxic properties and contribute to neuronal death. Additionally, alterations of antioxidant enzymes, such as SOD_1_, can increase the levels of free radicals and oxidative damage. It has also been suggested that the breakdown of the blood–brain barrier in this disease may contribute to neurodegeneration [42,43].

Neuroinflammation and oxidative stress are intimately linked in the pathogenesis of neurodegenerative diseases. Astrocytes and microglia potentiate the increases of free radicals, and activate motor neuron damage. While it has not yet been established whether neuroinflammation can be considered a primary process in disease generation, it is accepted that it plays roles in the progression and potentiation of other pathophysiological processes of ALS [38].

In conclusion, the neuropathological signature of ALS is shaped by processes such as oxidative stress, mitochondrial dysfunction, defective axonal transport, glutamate excitotoxicity, and neuroinflammation. Defects occur that influence free radical accumulation in motor neurons, including the loss of antioxidant function of enzymes (e.g., SOD_1_) and alterations in DNA repair. Oxidative stress can be increased by stimulation of NADPH oxidase activity, or disruption of mitochondrial respiratory chain activity due to impairment of mitochondrial dynamics. On the other hand, oxidative stress and glutamate excitotoxicity lead to alterations in apoptosis and the appearance of neuroinflammation in the surrounding microglia.

## 4. Pathogenic Risk Factors Involved in ALS

### 4.1. Neurodegeneration

Neurodegeneration is defined as a set of defective processes that lead to misfolded protein aggregates in the cytoplasm of motor neurons, generating oxidative and inflammatory damage, and leading to their death [43]. Due to the peculiar characteristics of motor neurons, ALS involves more rapid and progressive neurodegeneration than is observed in other neuron types. Motor neurons are large cytoskeletal cells that require high metabolic and mitochondrial activity. They are also characterized by the presence of easily oxidizable polyunsaturated fatty acids in their membrane [2]. Their high energy requirement and membrane characteristics make motor neurons particularly susceptible to the consequences of aberrant free radical accumulation. Free radicals cause lipid peroxidation, protein modifications, mitochondrial dysfunction, and DNA alterations in motor neurons, which lead to further accumulation of these toxic molecules, aggravating the progression of ALS neurodegeneration. Excess free radicals within the cell come from the mitochondria (via the respiratory chain), the endoplasmic reticulum, and peroxisomes. A portion of the free radicals is generated by enzymes, such as NADPH oxidase (NOX). These enzymes are found in motor neurons and neuroglia and are responsible for transferring electrons to oxygen and generating free radicals. Free radicals generated in the mitochondrial chain can induce the production of free radicals in NOX and vice versa, such that both mechanisms are potentiated, perpetuating neuronal damage. Moreover, these enzymes are regulated by SOD_1_, which is altered in both familial and sporadic types of ALS, thereby maintaining elevated oxidative stress [44]. This type of enzyme also exists in glia cells, which explains the role of neuroinflammation and how astrocytes can also generate free radicals that condition neurodegeneration. The NOX enzyme exists in several isoforms. Mice with ALS exhibit increased NOX type 2 enzyme, and its inhibition leads to an improvement of symptom progression [45,46].

In sporadic forms of ALS, oxidative stress can be generated by the interaction between several extrinsic factors, such as smoking or exposure to metals, which increase the prooxidant pathways, leading to accumulation of free radicals. This process, together with the susceptibility of motor neurons and their low capacity for renewal, causes motor neurons to be damaged and die, generating the symptoms and progression of this disease.

Neurodegeneration can start at any point of the pyramidal pathway. It can occur simultaneously in both upper and lower motor neurons, following the theory of mixed disease generation, which maintains that the disease appears as an independent process and originates in all motor neurons at the same time.

### 4.2. Relationship of Physical Activity Intensity and Muscle Metabolism

Van den Berg’s research group compared the lifestyles of 1557 individuals diagnosed with ALS in Europe versus 2922 healthy individuals [47]. Their findings showed that individuals diagnosed with ALS were more likely to have participated in intense exercise, with individuals who exercised more having an up to 26% higher risk of developing ALS compared with less-active individuals [47,48]. It has also been noted that certain professions, such as firefighters, soccer players [49], or military personnel [50], may be predisposed to ALS. An increased risk of ALS was even found with higher levels of leisure-time physical activity [51]. In this sense, the lack of association with occupational physical activity strengthens the hypothesis that a genetic profile or lifestyle that promotes physical fitness increases susceptibility to ALS rather than physical activity per se [51]. On the other hand, several articles analyze how the intensity of performed physical activity can induce oxidative stress initiated in the muscle fibers, which has repercussions on neurodegeneration of the motor neurons responsible for their innervation.

Skeletal muscle fibers are classified into fast-twitch (type IIa, IIb, and IIx) or slow-twitch (type I) muscle fibers, according to their functional and metabolic properties. In ALS, the motor neurons that innervate type IIb muscle fibers—i.e., the motor neurons that innervate rapidly fatiguing fibers responsible for anaerobic burst activity—are most vulnerable to the disease process [23].

The *SOD_1_*-mutated transgenic mouse model displays reduced contraction and loss of motor units in hindlimb muscles containing a high percentage (>90%) of type II muscle fibers. This corresponds to other findings showing that motor neurons innervating type II muscle fibers degenerate before slower fibers are affected [52]. Another study in *SOD_1_*-mutant mice reported altered muscle performance in both slow- and fast-twitch muscles, suggesting that muscle fiber vulnerability is a consequence of the type of motor neuron that innervates those muscle fibers, rather than the muscle metabolism [48].

The motor neurons most vulnerable to ALS pathogenesis are those that innervate muscle fibers with anaerobic metabolism. These muscle fibers are responsible for rapid contraction, fatigue more quickly, and are specialized for use in intense exercise—for example, in a 400 m sprint. This metabolism requires the attainment of a lot of energy, in the absence of oxygen, which is achieved using the anaerobic pathway of glucose degradation, producing energy and lactic acid. Lactic acid is mostly eliminated or oxidized by the muscles. Excess lactic acid can induce the transformation of a free radical that is not very harmful (superoxide radical) into another much more harmful free radical (perhydroxyl), due to the interaction of the superoxide radical with protons derived from lactic acid [53]. Individuals who perform intense physical exercise are more susceptible to rapid generation of lactate, which leads to increased oxidative stress, causing neurodegeneration and neuronal death in the neuromuscular unit and, consequently, in the motor neurons. A large number of studies show that free radicals play important roles as mediators of the muscle damage and inflammation produced due to strenuous exercise. Additionally, excess lactate can induce mitochondrial dysfunction in motor neurons, which generates greater oxidative stress [50]. These findings support that ALS generation would present a retrograde transmission, with its initial origin at the level of the motor unit. In this setting, the damage would begin in the lower motor neurons and progress to the upper motor neurons, which is how the onset of symptoms occurs in most cases of classical ALS. In early disease, the only symptoms are muscle weakness and atrophy, possibly reflecting damage in the lower motor neurons that innervate this type of muscle fibers. Progression of the disease leads to symptoms of upper motor neuron damage, such as spasticity and hyperreflexia.

The hypothesis that the intensity of performed physical activity may be a trigger for ALS is also supported by the fact that 52% of clinically validated ALS-related genes are differentially expressed after acute exercise, including the *C9ORF72* gene [54]. *C9ORF72* gene expression is downregulated during physical exercise, which could act synergistically by increasing toxicity in motor neurons. Genes linked to fibroblast growth factor (FGF) and nerve growth factor (NGF) signaling may also be altered. FGFs are highly expressed in motor neurons, and FGF secretion can be stimulated by oxidative stress, hypoxia, and hypovolemia, to induce astrocyte activation. Both produced by astrocytes, NGF and FGF have been shown to trigger motor neuron apoptosis under specific conditions in vitro, and this signaling has been implicated in ALS pathophysiology [54]. In fact, the progressive neuronal degeneration may be due to prolonged stimulation with FGF-1 or SOD-mediated oxidative stress in astrocytes [55]. It is possible that physical activity may be an ALS-triggering factor in patients with genetic susceptibility. For example, among individuals with a genetic alteration in *C9ORF72*, the ALS phenotype may be less aggressive in patients with a previous history of low physical activity, compared with patients having a history of strenuous physical activity [54]. Proposing that ALS may originate from the generation of oxidative stress in the neuromuscular junction, as a consequence of intense physical activity, allows us to develop recommendations for individuals genetically susceptible to ALS development, and to provide advice regarding their lifestyles and the type of physical exercise they should perform, with the aim of reducing their disease progression.

### 4.3. Intestinal Dysbiosis with Enteric Nervous System Involvement

The gastrointestinal tract harbors millions of microorganisms collectively referred to as the gut microbiota, which is shaped by host genetics and environmental exposure. Growing evidence supports the concept of the microbiota–gut–brain axis, through which the gut microbiota modulates part of the central and peripheral nervous system [56]. Based on this axis, it has been proposed that intestinal microorganisms can directly influence the central nervous system either through the production of neuroactive metabolites released into the systemic circulation, or through the enteric nervous system and cranial nerves, such as the vagus [57]. Therefore, changes in diet or infections can have negative or positive impacts on the manifestation of neuropathological and behavioral phenotypes of a disease.

Aging is accompanied by changes in the intestinal microbiota, which induce systemic inflammation [58]. Although there are not many scientific studies in humans, animal models have been particularly useful for demonstrating how aging affects the microbiota. For example, overgrowth of *Lactobacillus plantarum* is associated with a shortened lifespan due to the overproduction of oxygen free radicals affecting the enteric nervous system [59]. Additionally, the transplantation of fecal microbiota from young mice to aged mice has been performed, demonstrating protective effects of young mouse microbiota [60].

Gut microbial composition has been compared between ALS patients and healthy controls. Metagenomic sequencing of the gut microbiome has revealed a significantly different microbial composition in ALS patients compared with healthy controls, with ALS patients showing increased abundances of *Anaerostipes hadrus*, *Bacteroidales bacteria*, and *Bifidobacterium pseudocatenulatum*, and marginally decreased abundances of *Clostridium leptum* and *Escherichia coli* [61]. However, other studies show variable distributions of these bacteria, or even no significant differences in the intestinal microbial composition between ALS patients and healthy controls. These contradictory results from different studies may be at least partly due to the limited power of the studies, highlighting the need for systematic investigation of the microbiota in large cohorts of ALS patients and controls.

The possibility of microbiota alterations has also been examined in SOD_1_-mutant transgenic mice. Compared with wild-type littermate mice, SOD_1_-mutant mice exhibited a distinctly different microbiome composition, even before the onset of motor impairment, suggesting that alterations in the gut microbiome of SOD_1_ mice were not secondary to motor dysfunction [62]. It is evident that the generation of dysbiosis occurs prior to the appearance of symptoms. Additionally, SOD_1_-mutant mice display a longer intestinal transit time (from food ingestion until its elimination in feces), which coincides with weakness of the limb musculature [63]. This longer transit time reflects slow intestinal motility. Additional observations include altered intestinal proteins, including reduced smooth muscle myosin heavy chain (SMMHC) and increased glial fibrillary acidic protein (GFAP), indicating enteric damage [63] in the same way that the reactive astrocytes in the central nervous system (CNS) display hypertrophic nuclei and cell bodies and increased elaboration of processes with increased content of GFAP [55]. A recent study of manipulating the microbiome in ALS with metronidazole and clindamycin as antibiotics, and also with butyrate as a postbiotic, found reduced human-SOD1^G93A^ aggregation, decreased GFAP expression, and enhanced SMMHC expression both in the intestines and in the lumbar spine of SOD1^G93A^ mice with the antibiotics. With the postbiotic, these results were found in the intestines, and in the spine with reduced human SOD1^G93A^ aggregation and decreased GFAP expression. These results suggest that intestinal microbiome dysfunction may be correlated with the dysfunction of skeletal muscle activity and motor neuron in ALS [63].

The origin of ALS could lie in the connection between the enteric nervous system and the central nervous system. The cytoplasmic accumulation of phosphorylated TDP-43 (pTDP-43) aggregates in the CNS [64] of sporadic ALS (sALS) and most genetic ALS cases have been demonstrated within muscle [65] and peripheral nerve biopsies [66]. Surprisingly, a recent study on colonic biopsies [67] has also shown evidence of aggregation of pTDP-43 within the lamina propria (the mucosal connective tissue deep to the surface enterocytes) and the myenteric plexus. This raises the question of how pathology arising in the gut could reach the CNS, or even if this event would occur at the same time in both locations. The presence of pTDP-43 aggregates within intestinal tissues suggests the possibility of a similar mechanism of pathological spread to that observed in other neurodegenerative diseases [67]. Dysbiosis may induce neurodegeneration of the neurons of the enteric nervous system. The enteric nervous system is in contact with motor neurons that reach the spinal cord and cranial nerves, such as the vagus. Thus, intestinal dysbiosis could further support the retrograde theory of disease generation. The dysbiosis produced in SOD_1_-mutant mice included reduction of *Akkermansia muciniphila* compared with in wild-type littermates. Colonization of antibiotic-treated SOD_1_ mice with *A. muciniphila* prolonged their lifespan, and ameliorated their brain atrophy and motor deficits, as well as induced an increase of nicotinamide [62]. Nicotinamide is a form of vitamin B3, and part of the metabolic pathways that produce energy in cells. It is not yet known how this metabolite may curb ALS symptoms, but it could be linked to a reduction of oxidative stress in the nervous system and a modulation of mitochondrial genes in the spinal cord [68]. The protective effect of the bacterium *A. muciniphila* is likely due to the modulation of metabolites—in this case, nicotinamide—which are secreted from the blood system to the brain and protect motor neurons from neurodegeneration. The findings in transgenic mice have been verified with human microbiota mapping, which revealed that ALS cases had lower proportions of *A. muciniphila* and decreased nicotinamide, compared with cases without ALS [62].

Apart from metabolite production, mice with microbiota alterations also reveal the relationship with the immune system and a systemic response. Alterations in microglia may be strictly connected to the intestinal microbiota, since these mice have displayed aberrant and functionally impaired microglia, due to the generation of peripheral inflammation that activates microglia [69]. This reflects, for example, a significant reduction of butyrate-producing bacteria in SOD_1_-mutant mice. Butyrate-producing bacteria are known to play important roles in the control of intestinal inflammatory processes, and the maturation of the immune system, because the short-chain fatty acid butyrate inactivates lymphocyte maturation [70].

**Figure 3:**
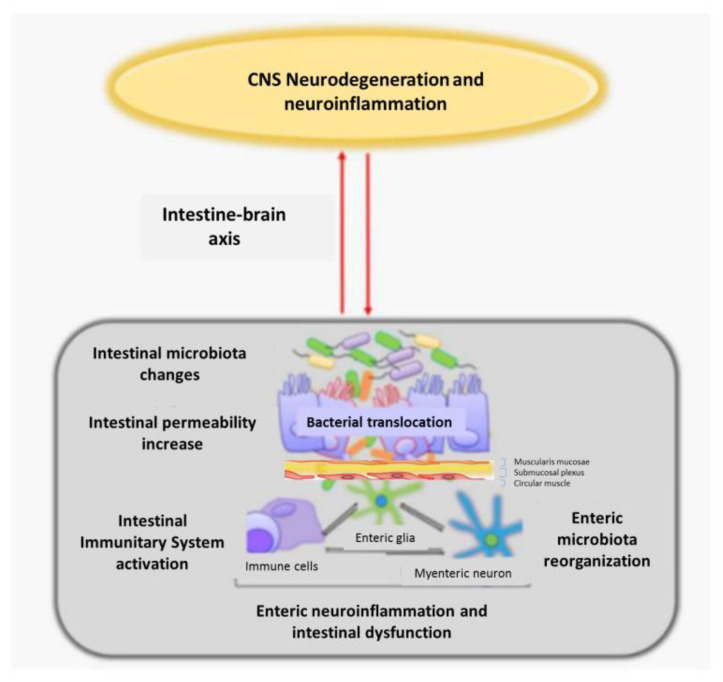
Relationship of alterations in the intestinal microbiota and enteric nervous system with neuroinflammation and neurodegeneration of the CNS, through the connection of the intestine-brain axis CNS: central nervous system (figure modified from [72]).

Mouse studies have provided evidence that microbiota changes may improve the disease phenotype (e.g., motor symptoms), although these findings remain purely experimental. Changes in the intestinal microbiota, impaired permeability, and systemic inflammation may represent the earliest processes that trigger ALS, suggesting that intestinal dysbiosis with enteric nervous system involvement may be a conditioning factor in ALS origin and a modulator of ALS progression [71] (Figure 3).

## 5. Conclusions and Future Remarks

Amyotrophic lateral sclerosis is the most frequent neurodegenerative motor neuron disease and has a devastating prognosis. We must obtain deeper knowledge of the pathophysiological mechanisms and the origin of this disease in order to achieve new diagnostic and therapeutic methods to improve the quality of life and survival of patients with ALS. Around 10% of ALS cases present as a familiar form with genetic alterations. The remaining 90% of ALS cases are sporadic and multifactorial in origin, likely involving a certain genetic susceptibility and association with various risk factors.

The pathophysiology of ALS includes alterations in RNA processing, protein metabolism and degradation, as well as defects in axonal transport of motor neurons, which lead to the presence of cytoplasmic protein clusters, inducing intracellular oxidative stress and neurodegeneration of motor neurons. Alterations of mitochondrial dynamics, neuroinflammation, and glutamate excitotoxicity aggravate oxidative damage in motor neurons, generating alterations of membranes, proteins, and DNA that lead to neuronal death. The existing anterograde theory proposes the primary involvement of upper motor neurons, due to hyperexcitability by glutamate. The mixed hypothesis suggests the possibility of other causes that synchronously affect both upper and lower motor neurons, such as neurodegeneration.

The retrograde propounds that ALS is precipitated by several factors associated with genetic susceptibility and intestinal dysbiosis with involvement of the enteric nervous system. All of these important insights are considerably useful for future clinical evaluations of this neurological disease.

Due to the great variety of possible causes involved in ALS etiopathogenesis, the pharmacological trials that are ongoing will determinate the next steps to follow for knowing more about this disease and for improving the patient’s prognosis and survival.

As in many other diseases in which there may be little effectiveness in treatment, it is necessary to indicate that it would be adequate to analyze those circumstances that precipitate its appearance, specifically the growing clinical interest of possible gene therapy, as well as the need to implement genetic studies in predisposed individuals.

## Data Availability

Not applicable.
